# Rosemary Essential Oil as a Natural Preservative in Fermented Thick Cream: Effects on Microbial Stability, Physicochemical Properties, and Sensory Quality

**DOI:** 10.1155/ijfo/2241675

**Published:** 2026-10-18

**Authors:** Karima Maaroufi, Baya Mhamdi, Chedia Aouadhi, Karim Hosni, Hichem Sebai

**Affiliations:** ^1^ Higher Institute of Biotechnology of Beja Laboratory Functional Physiology and Bio-resources Valorisation University of Jendouba Beja Tunisia uj.rnu.tn; ^2^ Higher Institute of Biotechnology of Beja Jendouba University Beja Tunisia; ^3^ University of Tunis El Manar (UTM) Pasteur Institute of Tunisia Laboratory of Epidemiology and Veterinary Microbiology. Group of Bacteriology and Biotechnology Tunis Tunisia utm.rnu.tn; ^4^ Laboratory of Natural Substances National Institute for Research and Physicochemical Analysis Sidi Thabet Tunisia

**Keywords:** antibacterial activity, essential oil, fermented thick cream, food preservation, rosemary

## Abstract

The present study evaluated the yield, phytochemical composition, antioxidant potential, and antimicrobial properties of *Salvia rosmarinus* essential oil (REO), as well as its potential application as a natural preservative in fermented thick cream. The essential oil obtained by hydrodistillation (yield: 0.9%) was analyzed by gas chromatography–mass spectrometry (GC–MS), which identified 15 compounds representing 100% of the total composition. The major constituents were 1,8‐cineole (34.26%), camphor (19.7%), and *α*‐pinene (14.27%). REO exhibited moderate antioxidant activity, with an IC₅₀ value of 1.12 mg/mL, indicating a notable free radical scavenging capacity. Antimicrobial assays demonstrated broad‐spectrum activity against both gram‐positive and gram‐negative bacteria, with inhibition zones ranging from 8 to 22 mm. The lowest minimum inhibitory concentration (MIC) values (0.5% v/v) were observed for *Bacillus cereus*, *Pseudomonas aeruginosa*, and *Streptococcus bovis*, whereas *Enterococcus faecium* showed the highest MIC (2% v/v), indicating strain‐dependent susceptibility. Incorporation of 0.13% REO into fermented thick cream improved sensory acceptance by enhancing flavor without causing overpowering intensity. Physicochemical and microbiological analyses showed delayed acidification and reduced microbial growth in REO‐treated samples compared with controls, confirming its preservative effect. Collectively, these findings highlight the multifunctional properties of REO as antioxidant activity and antimicrobial agent with acceptable flavor profile, supporting its potential application in food preservation and the development of natural functional ingredients.

## 1. Introduction

Functional foods enriched with bioactive components provide health benefits beyond basic nutrition and may contribute to reducing the risk of chronic diseases. Their increasing popularity reflects growing consumer interest in foods that promote well‐being and support a balanced lifestyle [1, 2]. Herbs and spices are particularly promising ingredients because, in addition to their sensory properties, they contain bioactive compounds with antimicrobial and antioxidant activities. These characteristics make them attractive natural alternatives to conventional food additives and suitable candidates for improving the quality and shelf life of functional foods [3–5].

Dairy products, which are rich in proteins, lipids, and essential micronutrients, represent suitable matrices for the incorporation of natural bioactive compounds [6]. Although the use of herbs, spices, and essential oils (EOs) has been investigated in several dairy products, their incorporation into fermented thick cream remains comparatively less explored. Fresh cream is a particularly interesting matrix because its high fat content may influence the partitioning and distribution of lipophilic compounds, whereas its emulsion structure can affect their availability and effectiveness. Moreover, the intense aroma of EOs may modify the characteristic sensory properties of cream [7, 8], making the selection of an effective concentration that remains acceptable to consumers a major challenge. Therefore, the activity of EOs observed *in vitro* cannot necessarily be extrapolated directly to complex dairy matrices [9].

EOs have attracted considerable interest as natural food preservatives because of their antimicrobial and antioxidant properties, while also contributing characteristic flavors and aromas [4]. Among them, *Salvia rosmarinus* (*Rosmarinus officinalis* L.) essential oil (REO) has demonstrated antibacterial, antifungal, and antioxidant activities, which are mainly associated with volatile constituents such as 1,8‐cineole, camphor, and *α*‐pinene [10–12]. It should be noted that phenolic compounds such as carnosic acid, carnosol, and rosmarinic acid are predominantly associated with rosemary extracts rather than the EO fraction and should therefore not be considered major constituents of REO [10–12].

Despite the documented biological activities of REO, its application in dairy products has been investigated more extensively in matrices such as yogurt, cheese, and other fermented products [13–15] than in fermented thick cream. The specific characteristics of cream, particularly its high lipid content, complex emulsion structure, and sensitivity to flavor changes, may influence the behavior and effectiveness of EO [16]. Consequently, evaluating REO directly in fermented thick cream is necessary to determine whether its *in vitro* bioactivity can translate into meaningful preservative effects without compromising product acceptability. Such an approach may provide useful insights into the practical application of REO in a high‐fat dairy matrix.

Therefore, this study is aimed at investigating the potential of REO as a natural bioactive additive in fermented thick cream. Specifically, the study sought to (i) characterize the chemical composition of REO, (ii) evaluate its antioxidant and antibacterial activities, and (iii) assess its effects when incorporated into fermented thick cream, with particular emphasis on physicochemical properties, microbial quality, and sensory acceptability during refrigerated storage. We hypothesized that REO incorporation could contribute to microbial control and overall product protection through its bioactive properties while maintaining acceptable sensory characteristics.

## 2. Materials and Methods

### 2.1. Plant Material and EO Extraction

The plant material of *S. rosmarinus* (formerly *R. officinalis*) was collected in the region of Aïn Draham (North‐West Tunisia, Kroumirie) during the spring season (March–April), corresponding to the preflowering stage. This area is characterized by a humid Mediterranean climate, light and well‐drained soils, and full sun exposure, which are optimal conditions for the biosynthesis of secondary metabolites and the preservation of EO quality.

EO isolation was performed according to the standard hydrodistillation method with a Clevenger apparatus, as reported in previous studies [17]. Approximately 100 g of dried leaves were placed in 600 mL of distilled water and distilled for 3 h. The resulting EO was separated from the aqueous phase, dried over anhydrous sodium sulfate, and stored in dark vials at 4°C until further analysis. The extraction yield (%) was determined from a single extraction and calculated as the ratio of the dry weight of the obtained EO to the dry weight of the initial plant material, expressed as a percentage.

### 2.2. Gas Chromatography–Mass Spectrometry (GC–MS) Analysis of EO

The chemical composition of *S. rosmarinus* EO was analyzed by a GC–MS HP 6890 (II) coupled with a HP 5973 mass spectrometer (Agilent Technologies, Palo Alto, California) operating in electron impact ionization mode (70 eV). A HP‐5MS capillary column (60 m × 0.25 mm i.d., 0.25 *μ*m film thickness) was used. The column temperature was programmed to rise from 40°C to 280°C at a rate of 5°C/min. The carrier gas was helium with a flow rate of 1.2 mL/min. The injector temperature was 250°C; the injection volume was 0.5 *μ*L, and the split ratio was 1:20. Scantime and mass range were 1 s and 50–550 m/z, respectively.

The volatile compounds were identified by comparison of their retention indices relative to (C_7_–C_20_) n‐alkanes with those of literature and/or with those of authentic compounds available in our laboratory and by matching their mass spectral fragmentation patterns with corresponding data (Wiley 275.L library) and other published mass spectra [18].

### 2.3. Antioxidant Activity–DPPH Radical Scavenging Assay

The antioxidant activity of the EO was evaluated using the 2,2‐diphenyl‐1‐picrylhydrazyl (DPPH) radical‐scavenging assay, according to Hatano et al. [19]. The DPPH solution was prepared at a concentration of 10^−4^ M in methanol. REO was dispersed in methanol prior to the assay, and no emulsifier was used. REO samples were tested at concentrations ranging from 0.5 to 5 mg/mL and mixed with the DPPH solution. After incubation for 30 min in the dark at room temperature, the absorbance was measured at 517 nm. A methanol blank was included to correct for the background absorbance of the solvent. The percentage of DPPH radical‐scavenging activity was calculated, and the IC₅₀ value was determined from the dose–response curve.

### 2.4. Antibacterial Activity

#### 2.4.1. Microorganisms

The EO was evaluated against eight bacterial reference strains, including *Escherichia coli* ATCC 8739, *Salmonella typhimurium* NCTC 6017, *Staphylococcus aureus* ATCC 29213, *Pseudomonas aeruginosa* ATCC 27853, *Listeria monocytogenes* ATCC 7844, *Bacillus cereus* ATCC 1247, *Enterococcus faecium* ATCC 19434, and *Streptococcus bovis ATCC* 49133.

#### 2.4.2. Disc Diffusion Assay

Antibacterial activity was evaluated using the disc diffusion method according to Aouadhi et al. [20], with minor modifications. Bacterial suspensions were standardized to approximately 1 × 10^8^ CFU/mL and uniformly spread onto tryptone soy agar (TSA) plates. Sterile paper discs (9 mm in diameter) were loaded with 15 *μ*L of EO and placed on the inoculated agar surface. Gentamicin was used as a positive control, whereas discs containing dimethyl sulfoxide (DMSO) alone served as the negative control. The plates were maintained at 4°C for 1 h to facilitate the diffusion of the EO constituents into the agar matrix. After incubation at 37°C for 24 h, the diameters of the inhibition zones were measured in millimeters.

#### 2.4.3. Determination of Minimum Inhibitory and Bactericidal Concentrations

Minimum inhibitory concentration (MIC) and minimum bactericidal concentration (MBC) values were determined using the broth dilution method according to Aouadhi et al. [20], with minor modifications. Bacterial suspensions were standardized to approximately 1 × 10^5^ CFU/mL and exposed to EO concentrations ranging from 0.07% to 50% (v/v). Because of the hydrophobic nature of the EO, DMSO was used as a dispersing solvent, and the same concentration of DMSO was included in the corresponding negative controls. Appropriate growth and sterility controls were also included. The tubes were incubated at 37°C for 24 h. The MIC was defined as the lowest EO concentration showing no visible bacterial growth. For MBC determination, aliquots from the nonturbid tubes were subcultured onto TSA, and the MBC was defined as the lowest EO concentration resulting in a ≥ 99.9% reduction in the initial bacterial inoculum.

### 2.5. Production of Fermented Thick Cream Flavored With EO

Fresh cream was pasteurized at 95°C for 5 s and subsequently inoculated with a commercial lactic acid bacterial starter culture at a concentration of 2% (v/v**)**. REO was added after pasteurization and immediately after starter inoculation, before fermentation at four concentrations: 0.068, 0.13, 0.24, and 0.34% (v/v). The inoculated cream was then fermented at 37°C for 7 h, until a final pH of approximately 4.7 was reached. Following fermentation, the products were packaged in plastic containers and stored at 4 ± 1°C under refrigerated conditions. Samples were analyzed after 1, 7, 14, 21, 28, and 35 days of storage. The different REO concentrations were tested to determine the level providing a desirable rosemary aromatic profile while maintaining the characteristic sensory properties of the fermented thick cream, including texture, flavor, and aroma.

The control and REO‐treated cream samples were prepared from the same batch of cream and processed under identical conditions, including inoculation, packaging, handling, and storage. The control sample did not receive REO, whereas the experimental samples were supplemented with REO at the specified concentrations. Thus, the absence or presence of REO was the only experimental variable distinguishing the control from the treated samples.

### 2.6. Sensory Evaluation of Rosemary‐Flavored Fermented Thick Cream

To determine the optimal dosage of REO and assess its impact on the sensory quality of fermented thick cream, a structured sensory evaluation was conducted. The sensory panel consisted of 20 participants, including 10 trained industry experts and 10 untrained consumers. The characteristics of the sensory panel are summarized in Figure 1. All participants were informed about the purpose and procedures of the sensory evaluation, and informed consent was obtained prior to participation.

**Figure 1:**
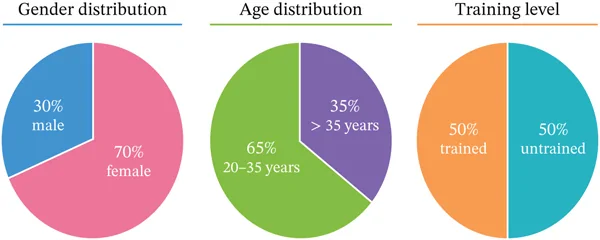
Characteristics of the sensory panel (*n* = 20).

Participants evaluated fermented thick cream formulations containing different concentrations of REO. The evaluation criteria included aromatic intensity (perceived strength of the rosemary aroma), flavor balance (harmony between the cream base and the rosemary aroma), overall acceptability (general liking and preference), sensory defects (e.g., bitterness or off‐flavors), and organoleptic attributes, including color, odor, texture, and mouthfeel. Samples were identified using random three‐digit codes and presented in a randomized order under controlled sensory conditions, with panelists blinded to the formulation identity. Water was provided between samples to cleanse the palate and minimize carry‐over effects.

Sensory attributes were evaluated using a 5‐point intensity scale ranging from 0 (*not perceived*) to 5 (*very high intensity*). Descriptive evaluation was also used to characterize the sensory attributes of the samples and support the selection of the most acceptable REO concentration. Sensory scores were subjected to appropriate statistical analysis to determine significant differences among the formulations.

### 2.7. Physicochemical Analyses

The measurement of pH and titratable acidity was conducted following standard methods commonly applied to milk and dairy products, using a calibrated pH meter and titration with 0.1 N NaOH, respectively. Subsequently, the total dry matter (DM) and fat content of the fermented thick cream samples were determined. For fat determination, 5 g of cream were placed in a butyrometer, then 10 mL of sulfuric acid, 1 mL of isoamyl alcohol, and 5 mL of distilled water were added sequentially. The butyrometer was sealed, shaken for homogenization, then centrifuged at 1200 rpm for 7 min. Fat content was read directly from the scale. The fat content was expressed in grams per liter (g/L) or as percentage (%) [21].

### 2.8. Bacteriological Analyses of Fermented Thick Cream

Microbiological analyses were performed to detect and enumerate yeasts, molds, coliforms, and total aerobic mesophilic bacteria in fermented thick cream samples. For yeasts and molds, 1 mL of sample was placed in sterile petri dishes, overlaid with Sabouraud dextrose agar, gently mixed, and incubated at 25°C for 2–5 days. Coliforms were enumerated by introducing 1 mL of sample into sterile petri dishes, adding Violet Red Bile Lactose (VRBL) agar (45°C–50°C), and homogenizing by gentle rotation; plates were incubated either at 30°C for 1–3 days for total coliforms or at 44°C for 1–2 days for fecal coliforms. Total aerobic mesophilic bacteria were counted using decimal dilutions. One milliliter of each dilution (10^−1^ to 10^−4^) was transferred into sterile petri dishes, mixed with plate count agar (PCA), and incubated at 30°C for 72 h.

## 3. Statistical Analysis

All experiments were conducted using three independent biological replicates (*n* = 3). For storage experiments, data were analyzed using a two‐way analysis of variance (ANOVA), with treatment (FC‐REO and FC‐CONT) and storage time as the two fixed factors. The treatment and storage time interaction was also evaluated. When significant differences were detected, Tukey′s HSD test was used for post‐hoc multiple comparisons. Statistical significance was considered at *p* < 0.05. Results are expressed as mean ± standard deviation (SD).

## 4. Results and Discussion

This study investigated the potential of REO as a natural additive in fermented thick cream. Their chemical compositions, as well as its antioxidant and antibacterial activities, were thoroughly characterized. Furthermore, its effects on cream stability, microbial quality, and sensory attributes during storage were systematically evaluated. The results presented below summarize these findings.

### 4.1. EO Yield and Composition

The EO yield of *S. rosmarinus* obtained in this study (0.9% based on dry weight) falls within the AFNOR standards (0.5%–2%) [22]; however, it remains relatively low compared to values commonly reported in Mediterranean regions. In Algeria and Tunisia, yields typically range between 1.6% and 1.9% [23, 24], whereas in Morocco they may exceed 3% [12]. In contrast, our result is closer to those reported in Sardinia, where seasonal variations can lead to yields as low as 0.29% [25], as well as to lower values observed in certain regions of Morocco and Pakistan (approximately 0.5%–1%) [26, 27].

Several studies have demonstrated that EO yield is strongly influenced by multiple factors, including the phenological stage of the plant, geographical origin, climatic conditions (temperature and humidity), soil characteristics, and genetic variability [25, 28, 29]. These parameters can significantly affect both the quantity and quality of the extracted oil. The chemical composition of REO also showed notable variability compared with previously published data. In the present study, 1,8‐cineole (34.26%), camphor (19.70%), and *α*‐pinene (14.27%) were identified as the major constituents (Table 1). In contrast, REO from Siliana (Tunisia) was characterized by higher levels of 1,8‐cineole (54.6%) and borneol (9.66%) [10], whereas Polish samples exhibited a more balanced composition, with *α*‐pinene (22.64%), camphor (21.84%), and 1,8‐cineole (21.53%) [30]. Oils from Tuscany (Italy) were dominated by *α*‐pinene (51.2–74.7%) [11], whereas Moroccan rosemary showed a profile rich in 1,8‐cineole (43.8%) and camphor (18.7%) [12].

**Table 1:** Chemical composition of *S. rosmarinus* L. essential oil.

Compound class	Volatile compounds	Retention indices	percentage (%)
Monoterpene hydrocarbons	Tricyclene	928	0.15
	*α*‐thujene	932	0.07
	*α*‐pinene	939	14.27
	Camphene	953	6.44
	*β*‐pinene	978	4.96
	*β*‐myrcene	988	0.49
	*Δ*‐3‐carene	1011	3.49
	*p*‐cymene	1026	3.13
	Limonene	1030	3.41

Oxygenated monoterpenes	1,8‐cineole	1031	34.26
	Camphor	1145	19.70
	Isoborneol	1159	1.52
	Borneol	1165	5.16

Sesquiterpenes and derivatives	*β*‐caryophyllene	1418	1.43
	Caryophylleneoxide	1569	1.52

Such compositional variability highlights the ecological plasticity of rosemary and supports the concept of distinct chemotypes influenced by environmental and genetic factors. However, this study is limited to a single harvest period and geographical origin, which may restrict the generalizability of the results. Future investigations integrating environmental parameters such as soil composition, altitude, and climatic conditions would provide deeper insight into the determinants of EO yield and chemical diversity.

### 4.2. Antioxidant Activity

In our study, REO exhibited an IC₅₀ value of 1.12 mg/mL (1120 *μ*g/mL), indicating moderate radical‐scavenging activity compared with the positive control, butylated hydroxytoluene (BHT), which exhibited an IC₅₀ value of 30 ± 0.01 * μ*g/mL. This value is lower than that reported by Ouibrahim [31] for samples from El Kala (3090 *μ*g/mL), indicating a stronger radical‐scavenging potential than those samples. However, our IC₅₀ is considerably higher than that obtained by Makhloufi [32] in Béchar (84.55 *μ*g/mL) and Kadri et al. [33] (110.20 *μ*g/mL), suggesting a weaker radical‐scavenging activity compared with these studies. Notably, the value reported by Kadri et al. [33] is close to that of the synthetic antioxidant BHT (40.5 *μ*g/mL), highlighting the high radical‐scavenging capacity reported for certain rosemary chemotypes. These comparisons indicate that the antioxidant activity of REO can vary considerably depending on geographic origin, environmental conditions, and chemical composition, supporting the possible influence of chemotypic variation.

Within the Lamiaceae family, rosemary is recognized as an important source of bioactive compounds with antioxidant potential. Its phytochemical constituents, including carnosol, carnosic acid, ursolic acid, rosmarinic acid, and caffeic acid, have been extensively investigated for their antioxidant and other biological activities [34]. However, it is important to note that these phenolic diterpenes and phenolic acids are mainly associated with rosemary extracts rather than with the EO fraction, which is predominantly composed of volatile compounds such as 1,8‐cineole, camphor, and *α*‐pinene. Consequently, the radical‐scavenging activity observed in our EO may be related to its specific chemical composition and the combined effects of its volatile constituents. Although 1,8‐cineole, camphor, and *α*‐pinene have been reported to exhibit various biological activities, their individual contributions to the activity observed in the present study cannot be established because these compounds were not tested separately. Therefore, the observed radical‐scavenging effect should be considered a property of the REO as a whole rather than attributed to any single constituent. The predominance of oxygenated monoterpenes may contribute to this activity. However, this hypothesis remains tentative and warrants further investigation using individual compounds and/or defined combinations of the major constituents. Rosemary is nevertheless recognized as an important medicinal and culinary herb containing numerous bioactive compounds with antioxidant potential [34].

### 4.3. Antibacterial Activity

The present study demonstrated that REO inhibited all tested bacterial strains, with inhibition zones ranging from 8.0 mm (*E. faecium*) to 22.0 mm (*B. cereus*) (Table 2). These results confirm the broad‐spectrum antimicrobial potential of REO, in agreement with previous studies reporting its activity against both gram‐positive and gram‐negative bacteria [4]. MIC and MBC assays further supported these findings, with values ranging from 0.5 to 2.0% (v/v) for MIC and 1.0 to 4.0% (v/v) for MBC, depending on the bacterial strain. The lowest MIC values (0.5%) were observed for *P. aeruginosa*, *B. cereus*, and *S. bovis*, indicating high susceptibility, whereas *E. faecium* exhibited the highest resistance (MIC = 2.0%). These results suggest that bacterial sensitivity is strain‐dependent rather than strictly associated with gram classification.

**Table 2:** Antibacterial activity of *S. rosmarinus* essential oil against selected bacterial strains.

Bacterial strain	Inhibition zone (mm)		MIC (% v/v)	MBC (% v/v)
	EO	Gentamicin		
*Staphylococcus aureus* ATCC 29213	12 ± 0.5^a^	20 ± 0.0^b^	1.0 ± 0.0	2.0 ± 0.0
*Pseudomonas aeruginosa* ATCC 27853	18 ± 0.33^a^	17 ± 0.0^a^	0.5 ± 0.0	1.0 ± 0.0
*Bacillus cereus* ATCC 1247	22 ± 0.5^b^	18 ± 0.0^b^	0.5 ± 0.0	1.0 ± 0.0
*Salmonella typhimurium* NCTC 6017	17 ± 1^a^	20 ± 0.0^b^	0.75 ± 0.0	1.5 ± 0.0
*Listeria monocytogenes* ATCC 7644	16 ± 1^a^	23 ± 0.0^b^	0.75 ± 0.0	1.5 ± 0.0
*Escherichia coli* ATCC 8739	12 ± 0.5^a^	24 ± 0.0^b^	1.0 ± 0.0	2.0 ± 0.0
*Streptococcus bovis* ATCC 49133	20 ± 1^a^	21 ± 0.0^a^	0.5 ± 0.0	1.0 ± 0.0
*Enterococcus faecium* ATCC 19434	8 ± 0^a^	21 ± 0.0^b^	2.0 ± 0.0	4.0 ± 0.0

*Note:* Values are expressed as mean ± standard deviation (*n* = 3). The letters (a) and (b) denote significant differences between means. Means followed by different letters are significantly different (*p* < 0.05).

Abbreviations: EO, essential oil; MIC, minimum inhibitory concentration; MBC, minimum bactericidal concentration.

Although gram‐positive bacteria are generally considered more susceptible to EOs because of the absence of an outer membrane, the marked sensitivity of *P. aeruginosa*, a gram‐negative bacterium, may be related to differences in membrane permeability and the susceptibility of this strain to the complex mixture of compounds present in REO. Previous studies have suggested that lipophilic constituents of REO, including 1,8‐cineole and *α*‐pinene, can interact with bacterial membranes, increasing membrane permeability and affecting membrane integrity [35]. However, these compounds were not evaluated individually in the present study; therefore, their specific contribution to the observed susceptibility of *P. aeruginosa* cannot be confirmed. This finding is particularly noteworthy given the substantial intrinsic resistance of *P. aeruginosa* to many antimicrobial agents. The observed activity may consequently be associated with the specific phytochemical profile of REO and the combined effects of its bioactive constituents on the bacterial cell envelope and membrane‐associated functions.

The antimicrobial activity observed in this study may result from the combined action of multiple constituents present in REO rather than from a single compound. Major constituents such as 1,8‐cineole, camphor, and *α*‐pinene have previously been associated with antimicrobial effects, including interactions with bacterial membranes and alterations in cellular permeability [36, 37]. Nevertheless, these mechanisms are inferred from previous studies and cannot be directly attributed to individual constituents under the experimental conditions of the present study. The observed antibacterial activity should therefore be interpreted as a property of the REO as a whole, potentially resulting from additive or synergistic interactions among its constituents. Overall, these findings reinforce the potential of REO as a promising natural antimicrobial agent for food preservation and other potential applications, although further studies using isolated compounds or defined combinations would be required to clarify the contribution of individual constituents and the underlying mechanisms of action.

### 4.4. Effect of EO on the Quality of Fermented Thick Cream

In order to establish the optimal dose of REO for incorporation into fermented thick cream, it was essential to balance sensory attributes such as taste, aromatic intensity, and overall product consistency. Four EO‐enriched cream formulations were prepared. The control sample consisted of the same cream formulation processed under identical conditions but without REO. Sensory evaluation was then conducted to assess the influence of each formulation on cream quality.

#### 4.4.1. Sensory Analysis and Organoleptic Characteristics

The sensory evaluation results demonstrated that REO significantly influenced the sensory attributes of fermented thick cream (Figure 2). At the lowest concentration (0.068%), panelists reported acceptable scores for color, texture, and odor; however, aromatic intensity remained low, indicating an insufficient release of rosemary flavor at this dosage level. The 0.13% formulation achieved the highest overall sensory scores across all evaluated parameters, including taste, aroma, texture, and appearance, reflecting the best balance between flavor intensity and product acceptability (Figures 2 and 3). In contrast, higher concentrations (0.24% and 0.34%) resulted in excessive aromatic intensity, bitterness, and olfactory saturation, which negatively affected overall acceptability and slightly altered texture perception.

**Figure 2:**
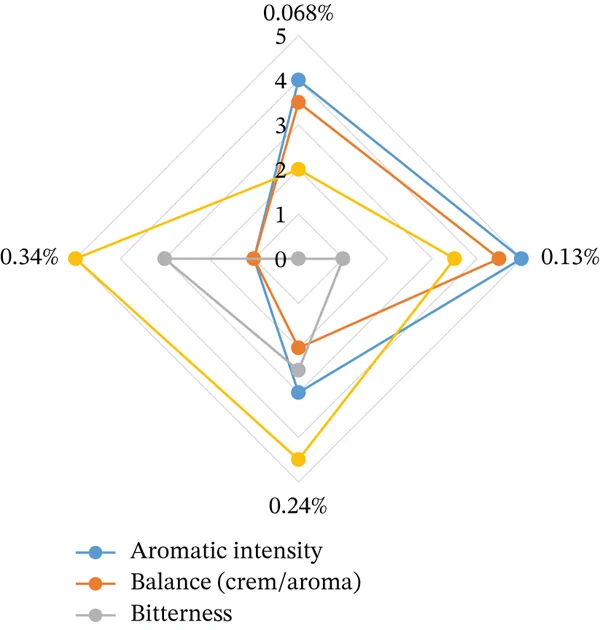
Sensory evaluation profile of fermented thick cream flavored with rosemary essential oil at different concentrations. Note: A score of 0 indicates very low satisfaction, whereas a score of 5 indicates high satisfaction.

**Figure 3:**
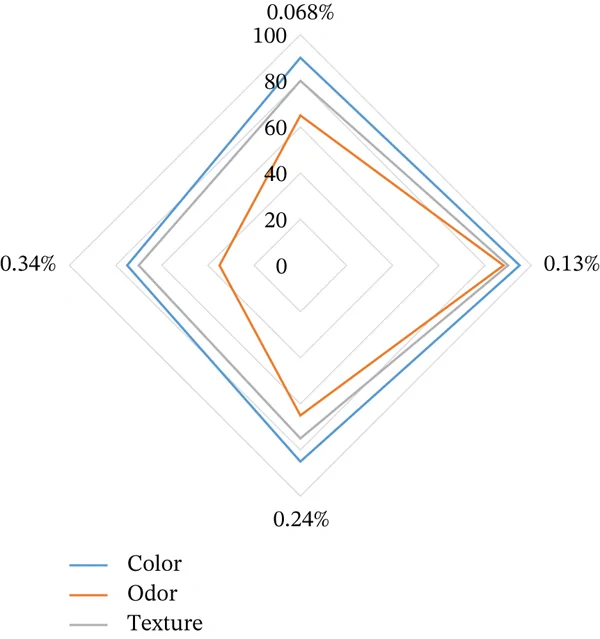
Percentage distribution of sensory acceptance levels for fermented thick cream formulated with rosemary essential oil at different concentrations.

These results confirm the dose–dependent effect of REO in dairy matrices, where low concentrations improve sensory quality, whereas excessive levels lead to flavor imbalance and reduced consumer preference [38]. The decrease in acceptability at higher concentrations may be explained by stronger interactions between volatile compounds of the EO and the lipid fraction of cream, which can enhance aroma perception and modify mouthfeel.

Overall, incorporation of REO at 0.13% was identified as the optimal formulation, ensuring a harmonious balance between aroma intensity, flavor acceptability, and sensory quality, highlighting the importance of precise dosage optimization in lipid‐rich food systems.

#### 4.4.2. Physicochemical Stability of Fermented Thick Cream During Storage

The incorporation of REO at 0.13% significantly influenced several quality parameters of fermented thick cream compared with the untreated control (Figure 4). The initial pH was slightly higher in the control sample than in the REO‐treated cream (4.70 vs. 4.67). Although the pH of both samples progressively decreased over the 35‐day storage period, presumably because of lactic acid production by lactic acid bacteria [39], the decrease was significantly less pronounced in the REO‐treated cream. At days 28 and 35, the pH decrease reached 0.27 and 0.45 units, respectively, compared with the control (Figure 4A). This stabilizing effect is consistent with the findings of Ali et al. [40], who reported that rosemary‐based additives can help maintain higher pH values in dairy products by limiting microbial proliferation. However, no significant effect on pH was observed following the incorporation of REO at concentrations of 0.6‰ and 0.13‰ in cheese [41], suggesting that the impact may depend on the type of dairy matrix and formulation conditions.

**Figure 4:**
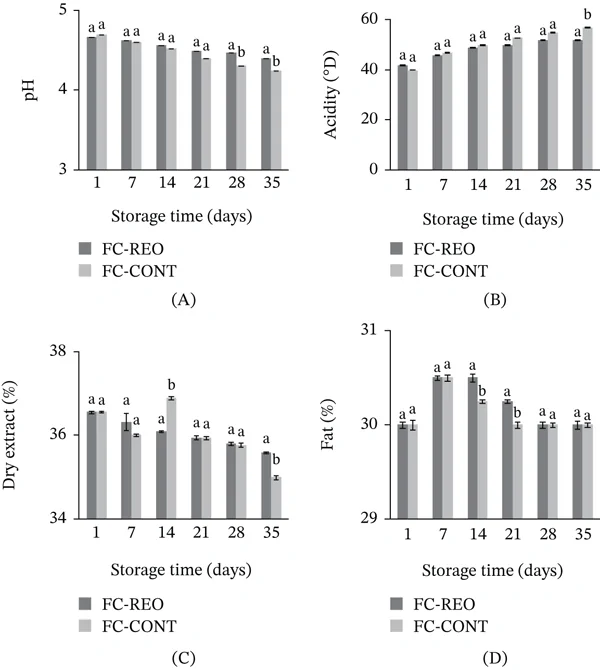
(A) pH variation. (B) Acidity variation. (C) Dry extract content. (D) Fat content concentration of fermented thick cream: control (FC‐CONT) and cream with rosemary essential oil (FC‐REO) over 35 days of storage. Values are means of three independent experiments. Means with different letters differ significantly (*p* < 0.05).

Similarly, titratable acidity increased progressively in both samples during storage, but to a greater extent in the control (40°D–57°D) than in the REO‐treated cream (42°D–52°D) (Figure 4B). This moderated increase may be attributed to the antimicrobial activity of REO, which can inhibit the growth and metabolic activity of acid‐producing microorganisms. It is important to note that although phenolic compounds such as rosmarinic acid, carnosic acid, and carnosol are often associated with strong antimicrobial and antioxidant effects, they are predominantly found in rosemary extracts rather than in the EO fraction. Therefore, the observed effects are more likely related to the activity of volatile constituents such as 1,8‐cineole, camphor, and *α*‐pinene. These compounds are known to disrupt microbial cell membranes and interfere with metabolic processes, thereby slowing acid production and spoilage. Comparable observations were reported by Noutfia et al. [41], who found that the addition of rosemary to cheese slightly affected acidity, although the effect was not statistically significant.

The DM content decreased progressively in both samples throughout storage, reflecting nutrient utilization by microorganisms and natural degradation processes. However, the REO‐treated cream exhibited greater stability, particularly during the later stages of storage, suggesting a protective effect against microbial and oxidative degradation. In contrast, the control sample showed a more pronounced decline, from 36.56% to 35% by day 35 (Figure 4C). These findings are consistent with those of Ghendov‐Moşanu et al. [42], who demonstrated that lipophilic plant extracts can help preserve DM in dairy matrices. Given that DM plays a crucial role in determining texture, viscosity, and overall product consistency [43], its stability is essential for maintaining the technological and sensory quality of dairy products [44].

Fat content showed a slight increase in both samples up to Day 14 (30.5% in REO‐treated cream vs. 30.25% in the control), followed by stabilization at approximately 30.0%, in agreement with previous findings [40] (Figure 4D). The fat fraction was not significantly affected by the incorporation of REO, which is consistent with the results reported by Noutfia et al. [41] and Himed‐Idir et al. [45], who also observed no significant changes in fat content following the addition of rosemary‐derived products.

Overall, these results demonstrate that REO supplementation can effectively delay acidification, moderate acidity development, and enhance the stability of DM without adversely affecting fat content. By limiting microbial growth and slowing biochemical changes, REO contributes to extending shelf life and preserving the physicochemical and sensory quality of fermented thick cream during refrigerated storage.

#### 4.4.3. Microbiological Analyses

REO significantly improved the microbiological stability of fermented thick cream during refrigerated storage compared to the control (Table 3). Both treatments exhibited comparable initial microbial loads (5.1 × 10^4^ CFU/mL), confirming similar starting quality. During storage, the REO‐treated cream (FC‐REO) showed a progressive reduction followed by stabilization of total viable counts, whereas the control sample (FC‐CONT) exhibited a typical spoilage pattern characterized by exponential microbial growth, reaching 3.5 × 10^6^ CFU/mL by Day 35. This marked divergence demonstrates the effectiveness of REO in inhibiting microbial proliferation and improving the microbiological stability of fermented thick cream under refrigerated conditions.

**Table 3:** Changes in total aerobic mesophilic bacteria, total coliforms, fecal coliforms, and yeasts and molds in fermented thick creams (Control = FC‐CONT and cream with REO = FC‐REO) over 35 days of storage. Values given are means of three independent experiments. Means with different letters differ significantly (*p* < 0.05).

Storage time (days)	Sample	Total aerobic mesophilic bacteria (log CFU/mL)	Total coliforms (log CFU/mL)	Fecal coliforms (log CFU/mL)	Yeasts and molds (log CFU/mL)
1	FC‐REO	4.70 ± 0.10^a^	< LOD	< LOD	< LOD
	FC‐CONT	4.70 ± 0.20^a^	< LOD	< LOD	< LOD
7	FC‐REO	4.30 ± 0.20^a^	< LOD	< LOD	< LOD
	FC‐CONT	5.00 ± 0.12^a^	< LOD	< LOD	< LOD
14	FC‐REO	3.95 ± 0.22^a^	< LOD	< LOD	< LOD
	FC‐CONT	5.60 ± 0.21^b^	< LOD	< LOD	< LOD
21	FC‐REO	3.78 ± 0.13^a^	< LOD	< LOD	< LOD
	FC‐CONT	5.85 ± 0.21^b^	< LOD	< LOD	< LOD
28	FC‐REO	3.00 ± 0.18^a^	< LOD	< LOD	< LOD
	FC‐CONT	6.00 ± 0.23^b^	< LOD	< LOD	< LOD
35	FC‐REO	2.88 ± 0.19^a^	< LOD	< LOD	< LOD
	FC‐CONT	6.54 ± 0.13^b^	< LOD	< LOD	< LOD

*Note:* < LOD: below the limit of detection (< 1 CFU/mL). Yeast, molds total and fecal coliforms were not detected throughout the storage period. The letters (a) and (b) indicate significant differences among means. Means followed by different letters are significantly different (*p* < 0.05).

Moreover, the absence of yeasts and molds in FC‐REO further supports the inhibitory effect of REO against spoilage‐associated fungi, which are commonly involved in the deterioration of dairy products during storage. This effect is particularly relevant for maintaining product quality and extending its microbiological stability. These findings are consistent with previous studies highlighting the preservative potential of REO in dairy systems. For instance, Nieto et al. [3] reported its broad‐spectrum antimicrobial activity, whereas Yeddes et al. [46] demonstrated a concentration‐dependent inhibitory effect against spoilage microorganisms. Similarly, Kloy et al. [47] and Kamel et al. [13] observed improved microbial stability and extended shelf life in dairy products supplemented with REO.

The stability of EOs and their bioactive constituents is an important consideration when evaluating their application as natural preservatives in food systems. Thermal exposure and storage conditions may influence the stability and functionality of plant‐derived oils and bioactive compounds [48, 49]. In the present study, however, REO was incorporated after pasteurization and was therefore not directly exposed to the pasteurization temperature (95°C for 5 s). Both the control and REO‐treated creams were subsequently subjected to identical fermentation, packaging, handling, and refrigerated storage conditions. Thus, the differences in microbiological stability observed between FC‐CONT and FC‐REO can primarily be associated with the presence of REO rather than differences in processing conditions. Nevertheless, changes in the composition or activity of REO constituents during fermentation and refrigerated storage cannot be excluded. Further studies assessing the stability of individual REO constituents throughout processing and storage would therefore be valuable for better understanding their contribution to the observed antimicrobial activity.

Interestingly, the optimal sensory concentration of REO in fermented cream (0.13% v/v) was lower than the MIC values determined in broth against the tested bacteria (0.5%–2.0% v/v). This apparent difference may be related to the distinct characteristics of the food matrix compared with the broth system. In cream, the high fat content and interactions between REO components and food constituents may affect the availability and antimicrobial activity of the EO. In addition, the complex background microbiota present in the cream differs from the pure bacterial cultures used in the MIC assay. Therefore, the antimicrobial effect observed at 0.13% in cream should not be directly compared with the MIC values obtained in broth. These findings highlight the importance of evaluating EO efficacy directly in the target food matrix.

REO stabilizes physicochemical parameters and microbial quality during storage because of its antioxidant and antimicrobial properties [50]. Its major monoterpenes, including 1,8‐cineole, camphor, and *α*‐pinene, exhibit notable antimicrobial activity [51]. This activity is based on disruption of cell membranes, enzymatic inhibition, and alteration of ion transport, reinforced by secondary components [52]. Antioxidant activity is also strongly associated with 1,8‐cineole and *α*‐pinene [53]. These mechanisms explain the ability of REO to simultaneously stabilize physicochemical parameters and microbial quality of foodstuffs during storage. Consequently, REO extends shelf life while preserving sensory and quality, confirming its relevance for food preservation [54, 55]. Moreover, the addition of REO improves oxidative and fermentative stability of cream‐cheese‐based flavored cheese, prevents lipid oxidation, and prolongs its shelf life [56]. Finally, its use limits *Salmonella* growth and reduces microbial spoilage in meat without affecting quality, with reductions in spoilage and pathogenic bacteria observed at both 4°C and 18°C [57].

From an industrial perspective, several additional factors should be considered before the practical application of REO in fermented cream, including its dispersion in the fat phase, emulsion stability, possible phase separation, flavor persistence during storage, packaging compatibility, batch‐to‐batch variation in EO composition, cost, consumer acceptance and labeling, and regulatory requirements. Further pilot‐scale studies are therefore needed to evaluate these aspects and to confirm the efficacy and stability of REO under industrial processing and storage conditions.

## 5. Conclusion

This study demonstrates that REO showed promising potential as a natural additive for fermented thick cream, offering multiple functional benefits. REO was characterized by a rich chemical profile dominated by 1,8‐cineole, camphor, and *α*‐pinene, and exhibited strong antioxidant and antibacterial activities. Its incorporation at 0.13% into fermented thick cream optimized sensory attributes, providing a harmonious aroma, balanced taste, and appealing texture. REO also enhanced physicochemical stability by moderating pH, acidity, DM, and fat content while significantly improving microbiological stability during storage by inhibiting bacterial, yeast, and mold growth. These findings highlight the potential of REO as a multifunctional natural preservative that can extend shelf life, may contribute to improved microbiological stability under the tested conditions, and enhance organoleptic quality in dairy products. This work not only confirms the bioactive properties of REO but also provides a practical framework for its application in functional dairy formulations, supporting sustainable alternatives to synthetic preservatives and contributing to innovation in the dairy industry.

**Nomenclature**
ATCC: American Type Culture Collection
DPPH: 2,2‐diphenyl‐1‐picrylhydrazyl
FC‐CONT: fermented thick cream–control
FC‐REO: fermented thick cream*–Rosmarinus* essential oil
GC/MS: gas chromatography–mass spectrometry
IZ: inhibition zones
MBC: minimum bactericidal concentration
MIC: minimum inhibitory concentration
REO: rosemary essential oil
RI: retention index

## Author Contributions

Karima Maaroufi: conceptualization, investigation, methodology, writing—original draft. Baya Mhamdi: data curation. Chedia Aouadhi: data analysis, validation, writing, review and editing. Karim Hosni: investigation, resources. Hichem Sebai: supervision, funding acquisition, project administration, review and editing.

## Funding

No funding was received for this manuscript.

## Ethics Statement

Formal ethical approval was not required for this study according to the applicable institutional procedures. All participants involved in the sensory evaluation were informed about the purpose and procedures of the study, and informed consent was obtained from all panelists prior to participation. The sensory evaluation was conducted voluntarily and in accordance with relevant ethical guidelines for research involving human participants.

## Consent

The authors have nothing to report.

## Conflicts of Interest

The authors declare no conflicts of interest.

## Data Availability

The data that support the findings of this study are available from the corresponding author upon reasonable request.
